# Multimodality imaging in diagnosing lipomatous atrial septal hypertrophy with atrial septal defect: a case report

**DOI:** 10.3389/fcvm.2023.1245213

**Published:** 2023-08-23

**Authors:** Yi Yu, Ming Ding, Jin-Lan Chen, Ting Wang, Yu-Han Chen, Xiao-Min Yang, Su-Yun Chen, Yue-Peng Wang, Yi-Gang Li

**Affiliations:** ^1^ Department of Cardiology, Xinhua Hospital Affiliated to School of Medicine, Shanghai Jiao Tong University, Shanghai, China; ^2^ Department of Radiology, Xinhua Hospital Affiliated to School of Medicine, Shanghai Jiao Tong University, Shanghai, China; ^3^ Department of Nuclear Medicine, Xinhua Hospital Affiliated to School of Medicine, Shanghai Jiao Tong University, Shanghai, China

**Keywords:** lipomatous atrial septal hypertrophy, atrial septal defect, multimodality imaging, transesophageal echocardiography, case report

## Abstract

**Background:**

Lipomatous atrial septal hypertrophy (LASH) with atrial septal defect (ASD) is a rare congenital anomaly. Although LASH is a histologically benign cardiac lesion characterized by excessive fat deposition in the interatrial septum that spares the fossa ovale, it has been associated with supraventricular arrhythmias or sick sinus syndrome. Application of multimodal imaging is crucial for accurate diagnosis, appropriate treatment of LASH with ASD, and follow-up.

**Case summary:**

A 68-year-old female patient presented with recurrent chest tightness and palpitation. Multimodal imaging revealed the characterizations of LASH and ASD. Two-dimensional transesophageal echocardiography showed a “dumbbell”-shaped involvement of the cephalad and caudal regions with sparing of a single secundum ASD. The septum with a brightness feature is an uncommon condition characterized by the deposition of unencapsulated fat cells in the atrial septum. Real-time four-dimensional transesophageal echocardiography reflected the lipomatous hypertrophy of the atrial septum and an oval-shaped ASD. Cardiac computer tomography angiography later confirmed this finding. The patient achieved a good clinical response with an ASD percutaneous occlusion guided by intracardiac echocardiography (ICE).

**Conclusion:**

This case demonstrates a LASH combined with ASD. Multimodality imaging can provide an accurate diagnosis and may guide the procedure for precise occlusion.

## Introductions

Lipomatous atrial septal hypertrophy (LASH) with atrial septal defect (ASD) is a rare abnormality. LASH is a histologically benign cardiac lesion characterized by excessive fat deposition in the interatrial septum that spares the fossa ovale (1–3). The prevalence rate of LASH was reported at approximately 2.2% in patients referred for a multislice computed tomography (CT) scan and 8% in patients undergoing transesophageal echocardiography (TEE) (1). Only a few cases of LASH were found to be related to hemodynamic alterations (congestive heart failure, superior vena cava obstruction), and surgical intervention should only be reserved for patients who show marked superior vena cava or right atrium obstruction (4–8). Although LASH has been associated with supraventricular arrhythmias (9, 10) or sick sinus syndrome (11), there are few reports in the literature on patients with both LASH and ASD (9, 12). Advances in multimodal imaging techniques may aid in diagnosing, treating, and following up LASH with ASD (13).

## Case presentation

A 68-year-old female patient with a 10-year history of hypertension was hospitalized in our department due to recurrent chest tightness and palpitation for half a month, accompanied by limb edema and dizziness. She had a history of multiple ground-glass nodules in both lungs, pulmonary bullae in the lower lobe of the right lung, esophageal papilloma, reflux esophagitis, gastric body submucosal eminence, and erosive gastritis. On presentation, she was afebrile, with a heart rate of 75 beats per minute, a blood pressure of 175/90 mmHg, and normal oxygen saturation.

An electrocardiogram documented that the patient had sinus arrhythmia and intermittent atrial premature beats, with short atrial tachycardia. The levels of serum N-terminal pro-B-type natriuretic peptide (NT-proBNP) (20.59 pg/ml) were elevated. The glycosylated hemoglobin level was 6.5%. Transthoracic echocardiography showed a secundum ASD with a size of 12 mm × 13.2 mm, and the interatrial septum was significantly thickened (19.4 mm). The right atrium and right ventricle were dilated, with a pulmonary-to-systemic flow ratio (Qp/Qs) of 2.0. There was mild tricuspid regurgitation, and the systolic pulmonary artery pressure was ≈49.8 mm Hg. The global left ventricle ejection fraction measured using the Simpson method was 63.4%, and grade I diastolic dysfunction was detected.

**Figure 1:**
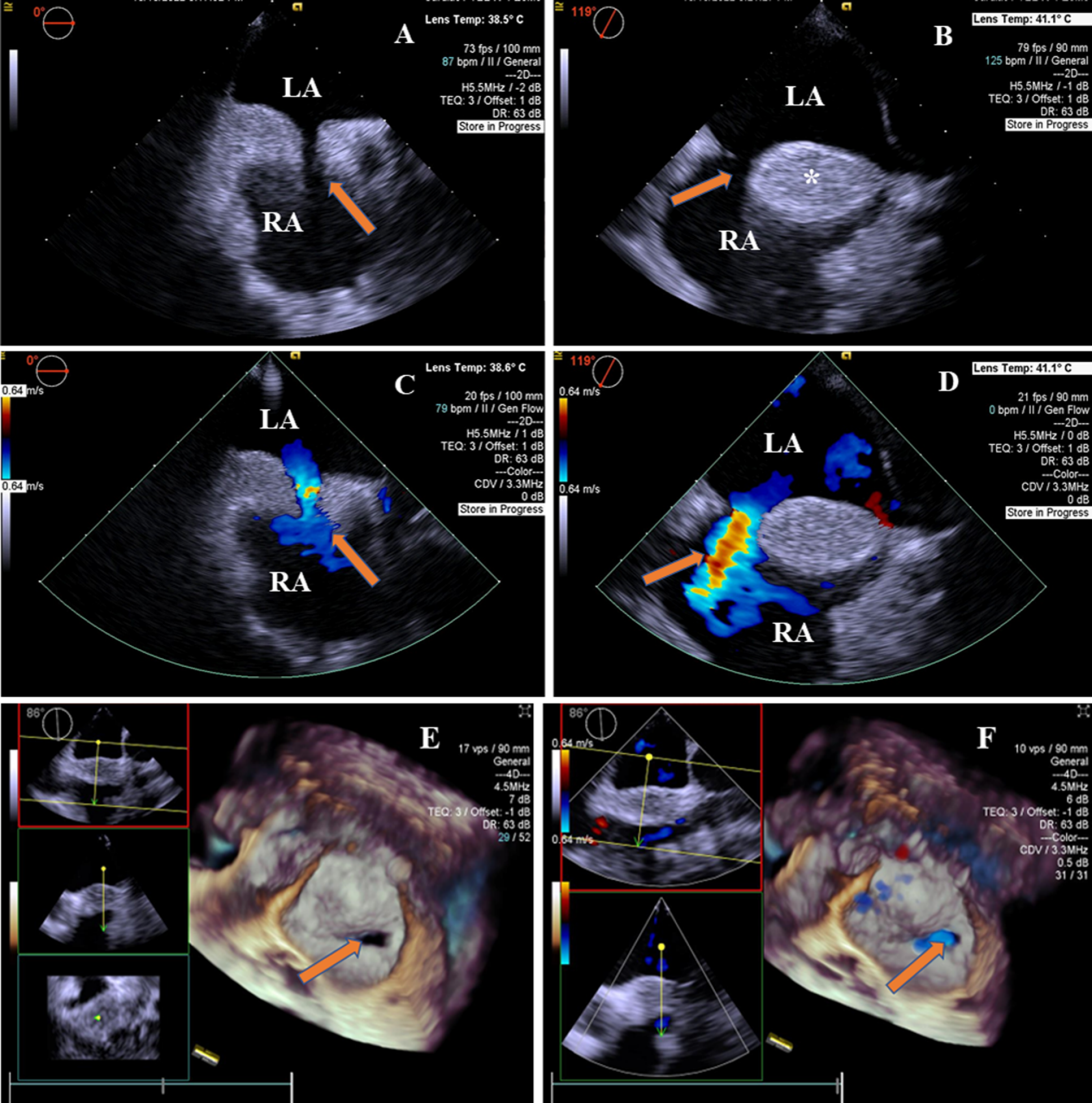
2D-TEE and 4D-TEE images revealed lipomatous hypertrophy of the atrial septum with atrial septal defect. (**A**) On 2D-TEE imaging, a single secundum ASD (arrow) and interatrial septum thickened to 20.7 mm in diameter were observed from 0° view. The septum with brightness features is characterized by the deposition of unencapsulated fat cells in the atrial septum. (**B**) 2D-TEE further documented a “dumbbell”-shaped involvement of the cephalad and caudal regions with sparing of the fossa ovalis (arrow) from 119° view. The interatrial septal fatty infiltration was demonstrated (*). (**C**) Color Doppler ultrasound of 2D-TEE showing a blood shunt from the left atrium to the right atrium (arrow) from 0° view. (**D**) Color Doppler ultrasound of 2D-TEE showing left-to-right shunt through ASD (arrow) from 119° view. (**E**) RT4D-TEE imaging reflected the whole ASD was surrounded by lipomatous hypertrophy of atrial septum (arrow) from 86° view. (**F**) Color Doppler ultrasound of RT4D-TEE showing blood flow of the ASD from the left to the right atrium (arrow). LA, left atrium; RA, right atrium.

Two-dimensional transesophageal echocardiography revealed a “dumbbell”-shaped involvement of the cephalad and caudal regions with sparing of a single secundum ASD with a size of 9 mm × 13.2 mm. The maximal thickness of the interatrial septum was 20.7 mm, and the thinnest atrial septum was 1.6 mm from the view at 0°–180°. The atrial septum presented with a brightness feature, a rare condition characterized by the deposition of unencapsulated fat cells in this area (Figures 1A,B). There was no obstruction in the superior and inferior vena cava. A two-dimensional color Doppler ultrasound evidenced a left-to-right shunt through ASD (Figures 1C,D). The diagnosis of LASH with ASD was established based on the above findings. Real-time four-dimensional transesophageal echocardiography (RT4D-TEE) confirmed the lipomatous hypertrophy of the atrial septum and an oval-shaped ASD (Figure 1E and Supplementary Movie I). In addition, a color Doppler ultrasound of RT4D-TEE demonstrated the defect appeared foraminal in a location with a significant left-to-right shunt, suggesting a “foraminal” or “fossa” ASD (Figure 1F and Supplementary Movie II).

**Figure 2:**
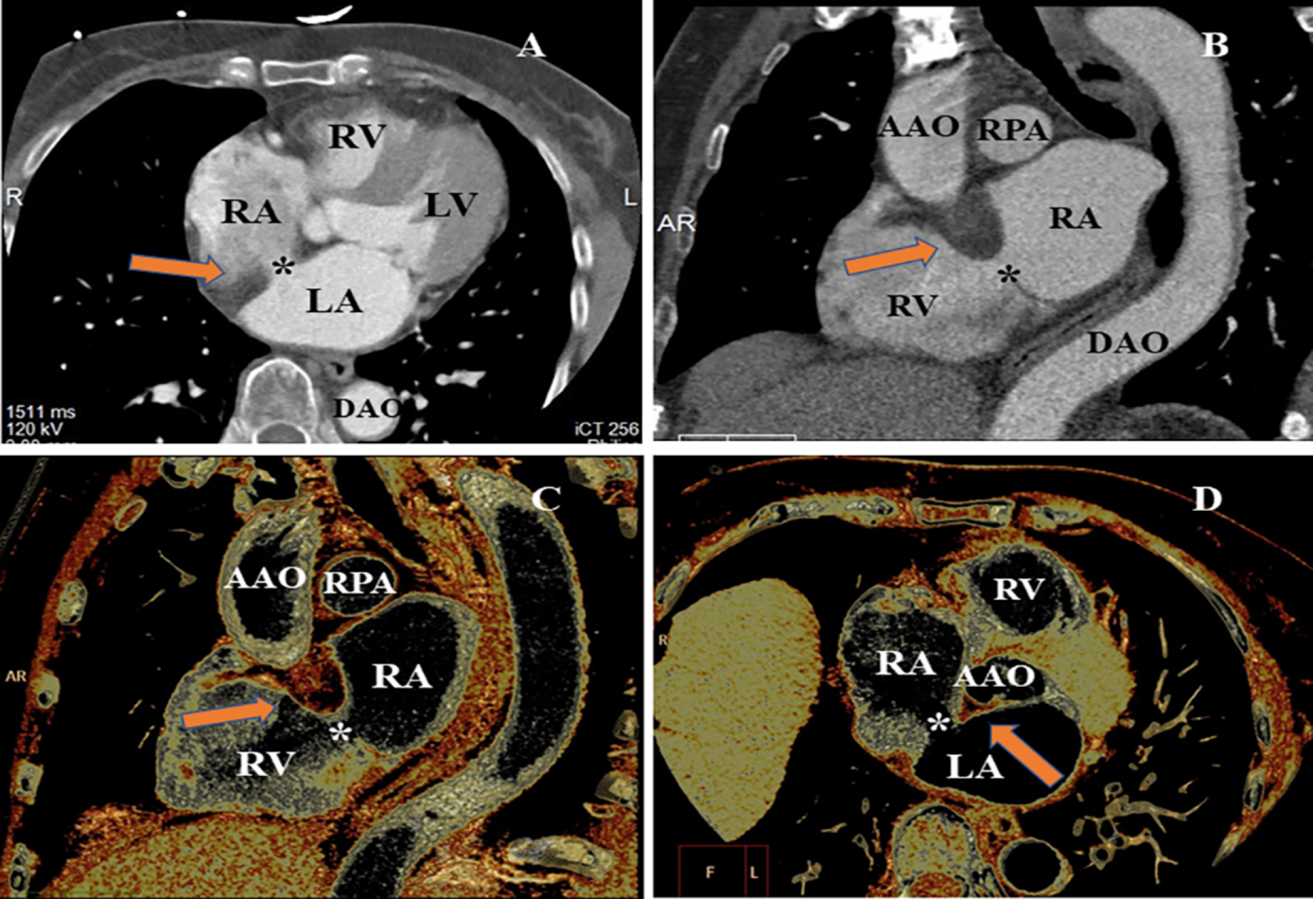
CCTA evaluating the LASH with ASD from different perspectives. (**A**) MPR image of CCTA found the loss of echogenicity between the left atrium and the right atrium (*). (**B**) MPR showed significant hypertrophy of the atrial septum (arrow), producing a dumbbell shape. (**C**) VR image indicated the thickened interatrial septum (arrow). (**D**) VR image of CCTA demonstrating the ASD and significant hypertrophy of atrial septum (arrow and *). MPR, multiplanar reconstruction; VR, volume rendering; LV, left ventricle; RV, right ventricle; RPA, right pulmonary artery; AAO, ascending aorta; DAO, descending aorta.

A cardiac computer tomography angiography (CCTA) scan was performed to further characterize the atrial septum and evaluate the status of the coronary artery. The CCTA images demonstrated a 9 mm × 13 mm defect on the atrial septum (Figures 2A,B). The volume rendering image of the CCTA scan showed a non-enhancing, smooth, well-marginated mass with an appearance similar to subcutaneous fat (Figures 2C,D). In addition, the CCTA scan revealed a myocardial bridge in the middle of the left anterior descending artery.

Myocardial perfusion imaging was performed to evaluate metabolic activity. Results showed no hypermetabolic lesions or abnormal myocardial blood perfusion in the heart. The left ventricular systolic and diastolic functions were normal.

**Figure 3:**
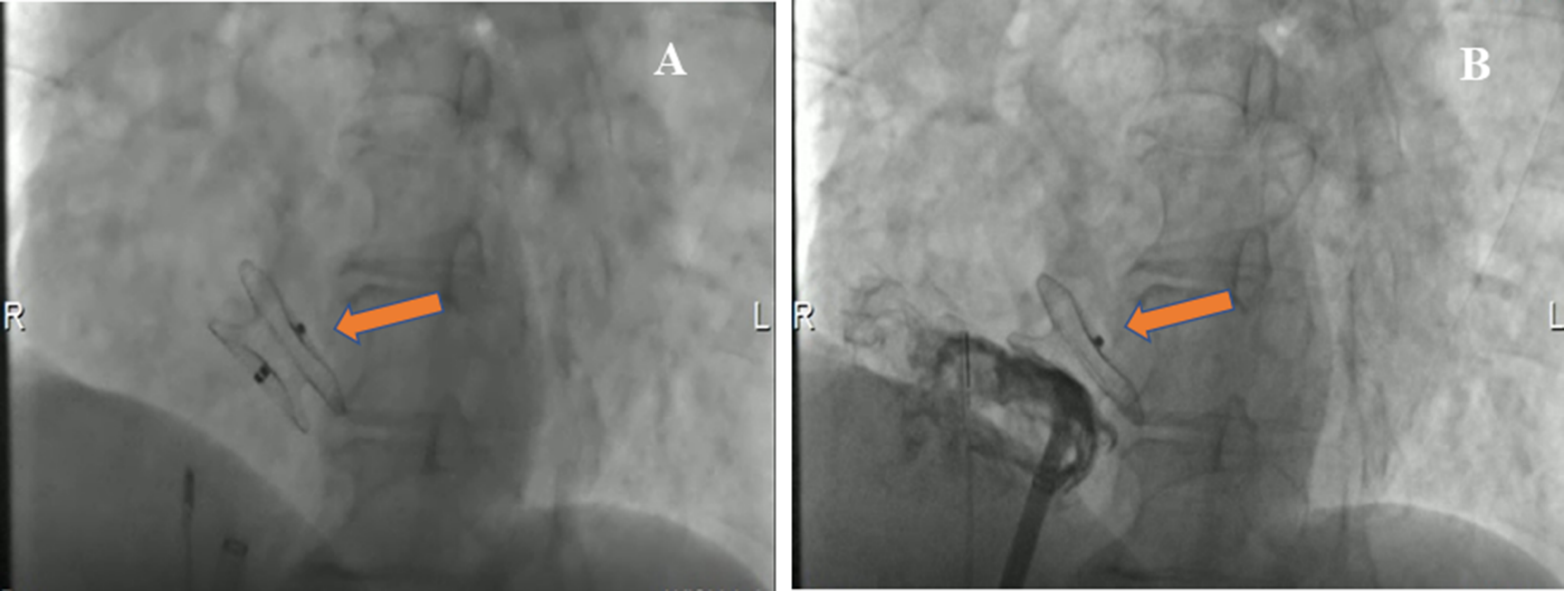
Cardioangiography (CAG) images demonstrating the closure with a Pushi septal occluder device. (**A**) CAG image visualized the position of a Pushi septal occluder device during the operation (arrow). (**B**) CAG image revealed the position of the septal occluder device was good, and there was no shunt between the left atrium and the right atrium after occlusion (arrow).

The patient underwent an ASD percutaneous occlusion under the guidance of intracardiac echocardiography (ICE) (Supplementary Movie III). During the operation, the LASH with ASD could be visualized by ICE from different angles. After balloon sizing, an 18 mm septal occluder device (Pushi, Shanghai) was successfully deployed (Figures 3A,B and Supplementary Movie IV). The occluder embraced the thick lipomatous cephalad rim and the thin “normal” caudal rim of the fossa ovalis. The left-to-right shunt disappeared, and no procedural complications, such as erosion or embolization, were observed. The patient was asymptomatic postoperation and followed up in an outpatient clinic (Supplementary Figures 1A–C). The atrial septum no longer increased in thickness. The systolic pulmonary artery pressure was ≈25.8 mmHg after ASD closure.

## Discussion

The patient was examined using multimodality imaging, including two-dimensional TEE (2D-TEE), RT4D-TEE, and CCTA. The diagnostic features of LASH with ASD include the following: (1) initial 2D-TEE imaging showed a “dumbbell”-shaped involvement of the cephalad and caudal regions with sparing of a single secundum ASD, (2) RT4D-TEE demonstrated lipomatous hypertrophy of the atrial septum and an oval-shaped ASD with a significant left-to-right shunt, and (3) CCTA images demonstrated the defect structure of the atrial septum and showed a non-enhancing, smooth, well-marginated mass with an appearance similar to subcutaneous fat.

The pathognomonic “dumbbell” shape is due to hypertrophy of the septum primum and secundum with sparing of the fossa ovalis (12). Echocardiographic features of LASH include a diffuse, echo-dense globular thickening anteroinferior or posterosuperior, and the magnitude of fat accumulation is >15 mm in thickness (14). As we all know, most cases of LASH are benign; nevertheless, patients with significant hypertrophy may develop obstruction of right atrial filling, dyspnea, or symptoms similar to congestive heart failure (15). A massive LASH larger than 20 mm may alter the nearby atrial musculature, leading to disturbed atrial conduction, resulting in arrhythmias (16–18). Heyer et al. (19) and Breuer et al. (6) reported that the obstruction of the right atrium by massive septal hypertrophy potentially requires surgical resection. In addition, older age and obesity are contributors to the pathogenesis of LASH; both are risk factors for the development of atrial fibrillation. However, there are few reports in the literature on patients with both LASH and ASD (9). Moir et al. (20) reported a case of successful percutaneous transcatheter closure using an FSO device for combined LASH and ASD with rim deficiency. In our case, 2D-TEE imaging indicated that LASH was associated with ASD, as evidenced by a left-to-right shunt flow signal on TEE. Finally, using 4D imaging, we confirmed the diagnosis of LASH combined with ASD, an uncommon condition illustrated as the deposition of unencapsulated fat cells in the atrial septum and oval hole.

In addition, the images could be misinterpreted as a tumor or other structural abnormalities (21–23). Masses in and near the interatrial septum may be either benign or malignant tumors. Kleiman et al. (22) reported a left atrial myxoma attached to the interatrial septum, increasing its thickness, a condition known as LASH. They then made an accurate diagnosis through TEE imaging. The interatrial septum was well visualized by echocardiography, although the image quality with TTE imaging is suboptimal compared with that of TEE imaging. If diagnosing a mass in or near the atrial septum is difficult, other available imaging modalities include cardiac magnetic resonance (CMR) imaging, myocardial perfusion imaging, and PET-CT. CMR imaging is useful in elaborating on imaging features, such as the location, shape, and signal intensity of LASH. It can reveal the presence of fatty tissues in the interatrial septum with the characteristic “dumbbell” shape and confirm the diagnosis. CMR can evaluate potential obstructions in the inflow of the right atrium and outflow of the right ventricle and accurately diagnose benign and malignant tumors (3, 24).

In this case, TEE played an important role in diagnosing LASH with ASD. Multiple sectional views at 0°–180° could be presented during TEE examinations (25), which help visualize LASH with ASD and exclude cardiac tumors. However, in patients with significant hypertrophy of the interatrial septum, diagnosing LASH with ASD by 2D-TEE may be technically challenging. Thus, it is crucial to further evaluate LASH with ASD in one cardiac cycle using RT4D-TEE imaging, which could provide some additional valuable information. By imaging in >1 RT4D-TEE planes, LASH can be seen and confirmed with an oval hole in the center of the atrial septum. In addition, RT4D-TEE offers a thorough evaluation of the interatrial septum. Thus, it can visualize the entire significant hypertrophy of the interatrial septum and ASD in the center of the septum. Meanwhile, the diagnosis of LASH should exclude atrial septal tumors. LASH has identified a uniform internal echo of the atrial septal tissue, distinct from the appearance of the neoplasm. Next, the high spatial resolution of CCTA has the advantage of visualizing significant hypertrophy of the atrial septum. ICE may provide additional information to aid a successful operation. It used to believe that catheter-based closure of ASD was contraindicated in patients with LASH (14). Lin et al. (12) reported a successful closure of ASDs in two patients with LASH using the Amplatzer muscular ventricular septal defect closure device. This device fitted well to the atrial septum and had no residual shunts at the 1-month follow-up. In our case, intraoperative ICE guidance combined with preoperative imaging examination results is safer, and the ASD occluder can be selected more accurately. To the best of our knowledge, this study is the first report of a successful percutaneous transcatheter closure using a Pushi septal occluder device for ASD combined with LASH.

In conclusion, in this case, the multimodality imaging techniques, especially for RT4D-TEE imaging, are pivotal for diagnosing a rare LASH with ASD and for deciding to perform the occlusion of ASD guided by ICE.

## Data Availability

The original contributions presented in the study are included in the article/**Supplementary Material**, further inquiries can be directed to the corresponding authors.
