# Supplementary figures and images for: Multimodality imaging in diagnosing lipomatous atrial septal hypertrophy with atrial septal defect: a case report
Source: Front Cardiovasc Med. 2023 Aug 23;10:1245213. doi: 10.3389/fcvm.2023.1245213 (PMC10482038; doi:10.3389/fcvm.2023.1245213)

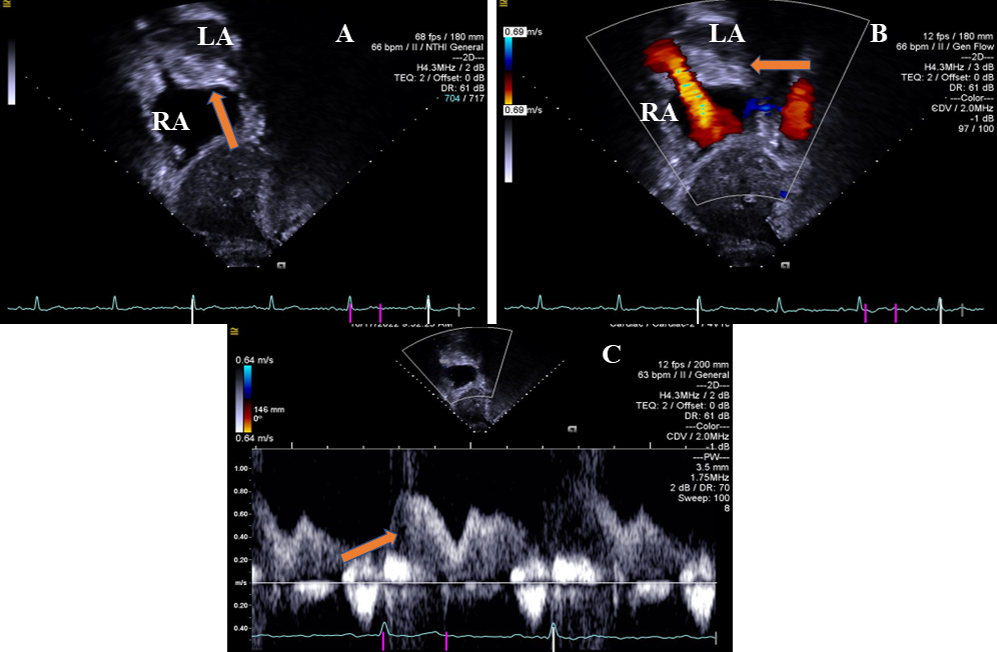

Supplement: Supplementary file 5 [file Image1.tif]

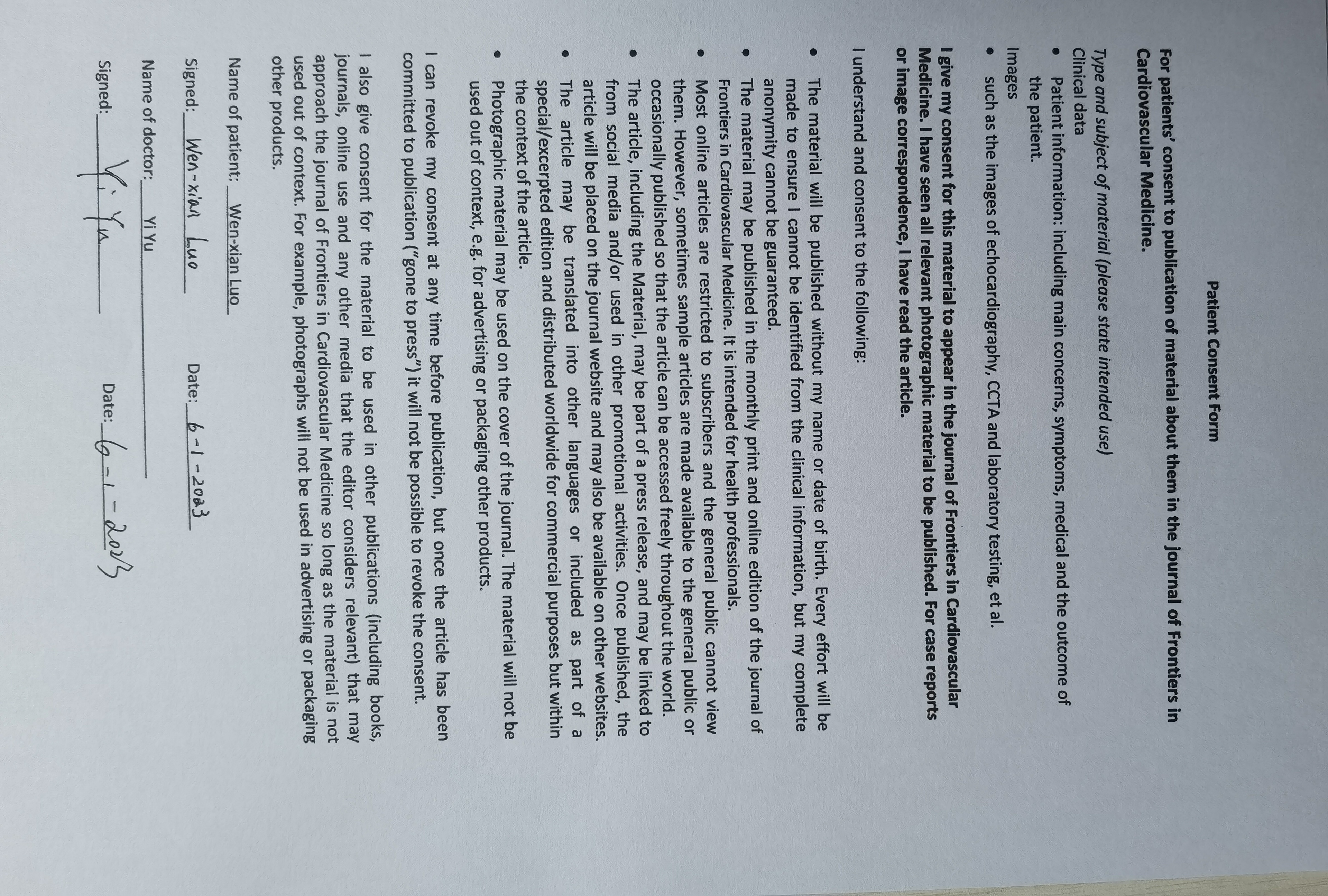

Supplement: Supplementary file 6 [file Image2.jpeg]

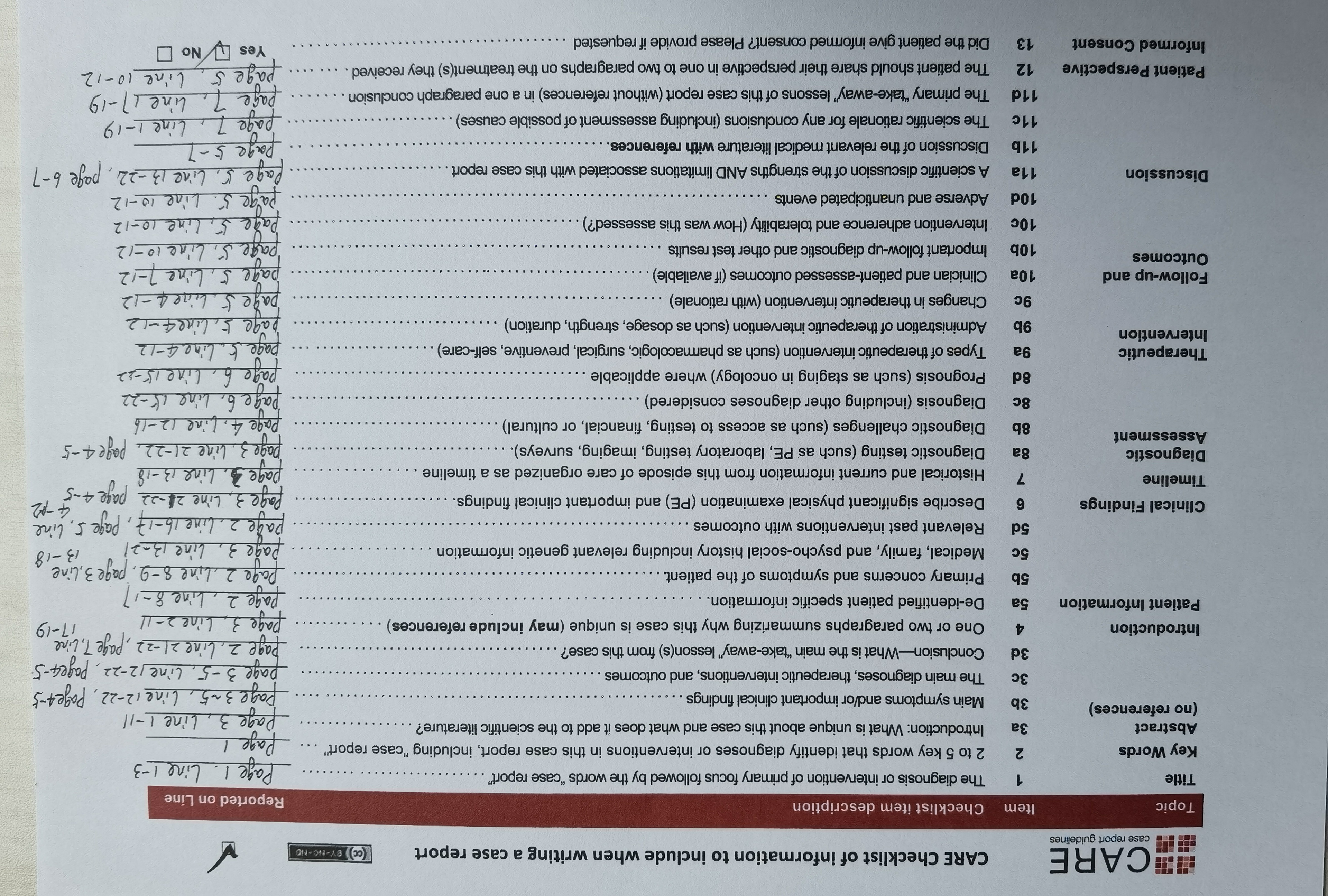

Supplement: Supplementary file 7 [file Image3.jpeg]
